# [Expression of Concern] miR-148a inhibits cell proliferation and migration through targeting ErbB3 in colorectal cancer

**DOI:** 10.3892/ol.2026.15655

**Published:** 2026-05-19

**Authors:** Wei Zhao, Jianbao Zheng, Guangbing Wei, Kui Yang, Guanghui Wang, Xuejun Sun

Oncol Lett 18: 2530–2536, 2019; DOI: 10.3892/ol.2019.10581

Following the publication of the above paper, it has been drawn to the Editor's attention by a concerned reader that, regarding the migration and invasion cell assay experiments shown in Fig. 3A (for the LoVo cell line) on p. 2534, the ‘mimic NC’ and ‘inhibitor NC’ data panels for the invasion assay panels (lower row) showed an overlapping section, such that these data panels, which were intended to show the results of differently performed experiments, had apparently been derived from the same original source.

The authors have been contacted by the Editorial Office to offer an explanation for this apparent anomaly in the presentation of the data in this paper, and we are awaiting their response. Owing to the fact that the Editorial Office has been made aware of this potential issue surrounding the scientific integrity of this paper, we are issuing an Expression of Concern to notify readers of this potential problem while the Editorial Office continues to investigate this matter further.

